# Glycemic control and cancer outcomes in oncologic patients with diabetes: an Italian Association of Medical Oncology (AIOM), Italian Association of Medical Diabetologists (AMD), Italian Society of Diabetology (SID), Italian Society of Endocrinology (SIE), Italian Society of Pharmacology (SIF) multidisciplinary critical view

**DOI:** 10.1007/s40618-024-02417-z

**Published:** 2024-06-27

**Authors:** A. Natalicchio, N. Marrano, M. Montagnani, M. Gallo, A. Faggiano, MC Zatelli, A. Argentiero, M. Del Re, S. D’Oronzo, S. Fogli, T. Franchina, D. Giuffrida, S. Gori, A. Ragni, G. Marino, R. Mazzilli, M. Monami, L. Morviducci, V. Renzelli, A. Russo, L. Sciacca, E. Tuveri, A. Cortellini, M. Di Maio, R. Candido, F. Perrone, G. Aimaretti, A. Avogaro, N. Silvestris, F. Giorgino

**Affiliations:** 1 Department of Precision and Regenerative Medicine and Ionian Area, Section of Internal Medicine, Endocrinology, Andrology and Metabolic Diseases, University of Bari Aldo Moro, Piazza Giulio Cesare, 11, I-70124 Bari, Italy; 2 Department of Precision and Regenerative Medicine and Ionian Area, Section of Pharmacology, University of Bari Aldo Moro, Bari, Italy; 3 Endocrinology and Metabolic Diseases Unit, Azienda Ospedaliero-Universitaria SS Antonio e Biagio e Cesare Arrigo of Alessandria, Alessandria, Italy; 4 Endocrinology Unit, Department of Clinical & Molecular Medicine, Sant’Andrea Hospital, Sapienza University of Rome, Rome, Italy; 5 Section of Endocrinology, Geriatrics and Internal Medicine, Department of Medical Sciences, University of Ferrara, Ferrara, Italy; 6 Medical Oncology Unit, IRCCS Istituto Tumori “Giovanni Paolo II”, Bari, Italy; 7 Department of Clinical and Experimental Medicine, University of Pisa, 55, Via Roma, 56126 Pisa, Italy; 8 Interdisciplinary Department of Medicine, University of Bari Aldo Moro, Bari, Italy; 9 Clinical Pharmacology and Pharmacogenetics Unit, Department of Clinical and Experimental Medicine, University of Pisa, Pisa, Italy; 10 Medical Oncology Unit, Department of Human Pathology “G. Barresi”, University of Messina, Messina, Italy; 11 Department of Oncology, Istituto Oncologico del Mediterraneo, Viagrande, Catania Italy; 12 Oncologia Medica, IRCCS Don Calabria-Sacro Cuore Hospital, Negrar, Verona Italy; 13 Internal Medicine Department, Ospedale dei Castelli, Asl Roma 6, Ariccia, Rome Italy; 14 Diabetology, Careggi Hospital and University of Florence, Florence, Italy; 15 Diabetology and Nutrition Unit, Department of Medical Specialties, ASL Roma 1 – S. Spirito Hospital, Rome, Italy; 16 Diabetologist and Endocrinologist, Italian Association of Clinical Diabetologists, Rome, Italy; 17 Department of Surgical, Oncological and Oral Sciences, Section of Medical Oncology, University of Palermo, Palermo, Italy; 18 Department of Clinical and Experimental Medicine, Endocrinology Section, University of Catania, Catania, Italy; 19 Diabetology, Endocrinology and Metabolic Diseases Service, ASL-Sulcis, Carbonia, Italy; 20 Operative Research Unit of Medical Oncology, Fondazione Policlinico Universitario Campus Bio-Medico, Via Alvaro del Portillo, 200, 00128 Rome, Italy; 21 Department of Medicine and Surgery, Universitá Campus Bio-Medico di Roma, Via Alvaro del Portillo, 21, 00128 Rome, Italy; 22 Department of Surgery and Cancer, Hammersmith Hospital Campus, Imperial College London, London, UK; 23 Department of Oncology, University of Turin, AOU Città Della Salute e della Scienza di Torino, Turin, Italy; 24 Department of Medical Surgical and Health Sciences, University of Trieste, 34149 Trieste, Italy; 25 Clinical Trials Unit, National Cancer Institute, Naples, Italy; 26 Endocrinology, Department of Translational Medicine, Università del Piemonte Orientale, Novara, Italy; 27 Department of Medicine, Section of Diabetes and Metabolic Diseases, University of Padova, Padua, Italy

**Keywords:** Diabetes, Cancer, Glycemic control, Glycemia, Cancer outcomes, Cancer progression

## Abstract

Background: Increasing evidence suggests that diabetes increases the risk of developing different types of cancer. Hyperinsulinemia, hyperglycemia and chronic inflammation, characteristic of diabetes, could represent possible mechanisms involved in cancer development in diabetic patients. At the same time, cancer increases the risk of developing new-onset diabetes, mainly caused by the use of specific anticancer therapies. Of note, diabetes has been associated with a ∼10% increase in mortality for all cancers in comparison with subjects who did not have diabetes. Diabetes is associated with a worse prognosis in patients with cancer, and more recent findings suggest a key role for poor glycemic control in this regard. Nevertheless, the association between glycemic control and cancer outcomes in oncologic patients with diabetes remains unsettled and poorly debated. Purpose: The current review seeks to summarize the available evidence on the effect of glycemic control on cancer outcomes, as well as on the possibility that timely treatment of hyperglycemia and improved glycemic control in patients with cancer and diabetes may favorably affect cancer outcomes.

## Introduction

The incidence of diabetes is rapidly spreading worldwide. In 2021, 537 million adults (20–79 years) were living with diabetes (9.2% of adults), over 90% of whom with type 2 diabetes (T2D). This number is predicted to rise to 643 million by 2030 and 783 million by 2045 [1]. Diabetes is often burdened by disabling comorbidities, such as cardiovascular and renal complications, that reduce the quality of life and life expectancy of the affected individuals [2]. Of note, diabetes and its complications were responsible for 6.7 million deaths in 2021 (1 every 5 s) [1]. Interestingly, in recent years, the advances in diabetes management and increase in the life expectancy of diabetic patients have made it possible to identify less-recognized and longer-term comorbidities, defined as emerging complications of diabetes, including cancer [3]. Of note, the increase in the incidence of diabetes is paralleled by the increasing incidence of cancer [4, 5]. Patients with diabetes, particularly T2D, are characterized by an increased risk of developing different types of cancer (especially bladder, breast, colorectal, endometrial, gallbladder, liver, and pancreatic cancers) and reduced survival after cancer diagnosis [6, 7]. At the time of cancer diagnosis, ⁓18% of patients have pre-existing diabetes, and it is estimated that approximately 20% of people with cancer have or will develop diabetes [8, 9], more than double the incidence of diabetes in the global adult population. The magnitude of risk between diabetes and cancer varies across cancer sites. For hepatocellular, pancreatic, and endometrial cancers, the increased risk associated with diabetes may be up to two-fold, whereas for other cancers, such as colon and breast, the relative risk increases are closer to 20–40% [10]. On the other hand, the evidence regarding the associations of T2D with other cancers such as kidney and lung cancer remains inconclusive [6, 11, 12]. The relationship between prostate cancer and diabetes is unique, since it is the only cancer where diabetes appears to be protective [13]. The coexistence of diabetes and cancer may be related to these as widespread pathologies making the probability of their occurrence in the same patient very high. Indeed, diabetes and cancer share many risk factors, such as obesity, sedentary lifestyle, unbalanced diet, cigarette smoking, and excessive alcohol consumption, which may further increase the likelihood of co-occurrence [12, 14]. Nevertheless, growing evidence suggests that the link between diabetes and cancer may be causal with these two pathological conditions triggering each other. For instance, many cancer cells overexpress insulin receptors, especially the pro-proliferative A isoform, and therefore are more responsive to the mitogenic effects of insulin [14]. In this context, the hyperinsulinemia typical of the early stages of T2D may stimulate cancer cells proliferation [10, 14]. Insulin may also promote carcinogenesis through indirect mechanisms, via reduction in circulating levels of insulin-like growth factor (IGF)-binding proteins, leading to excess IGF-1 and IGF-2, which further promote cancer cell proliferation [10, 15]. In addition, since glucose excess is an important source of energy for cancer cells (“Warburg effect”), hyperglycemia typical of diabetes could promote tumor growth [10, 12, 14, 16]. Hyperglycemia and insulin resistance may be also responsible for further increase in insulin secretion. Chronic low-grade inflammation characteristic of diabetes may also promote neoplastic transformation, cancer cell proliferation, and tumor spreading [14]. In addition, hyperinsulinemia, hyperglycemia, and inflammation can intensify the production of reactive oxygen species, therefore promoting oxidative stress [17], which is known to be a biological event able to trigger or enhance the tumorigenic process [14], especially when it involves tumor suppressor genes [18]. Recently, it has been suggested that several miRNAs, which mainly regulate the insulin signaling pathway, may be involved in the pathogenesis of both diabetes and cancer [19]. In addition, some endocrine disruptors derived from commonly employed compounds for manufacturing and processing, particularly polybrominated diphenyl ethers (PBDEs), may interfere with both metabolic and oncogenic pathways [20]. Lastly, over the years it has been hypothesized that some anti-diabetes drugs may be responsible for the increased risk of cancer in patients with diabetes. In 2009, 4 independent studies [21–24] suggested that exogenous insulin may be associated with an increased risk of cancer, although more recent epidemiological studies seem to refute this hypothesis [25–27]. Similarly, incretin drugs (GLP-1 receptor agonists [GLP-1RAs] and DPP-4 inhibitors [DPP-4i]) were initially associated with an increased risk of pancreatic and medullary thyroid cancers, although this association has not been confirmed in more recent studies [28–30]. In 2018, an increased risk of cholangiocarcinoma was reported in patients treated with DPP-4i [31]. Although this correlation remains to be validated, it is supported by the biological evidence that high levels of GLP-1 are associated with reduced apoptosis and increased proliferation of cholangiocytes [32, 33]. In 2011, the Food and Drug Administration issued a warning regarding the use of pioglitazone [34], after studies had shown an association between its use and a higher risk of bladder cancer [35]. Since then, numerous studies have been conducted and a recent meta-analysis reported a small but statistically significant increase in the risk of bladder cancer in patients treated with pioglitazone [36]. A similar risk has also been observed in patients treated with rosiglitazone [37]. Finally, preclinical studies have suggested that the use of SGLT2 inhibitors (SGLT2i) may be associated with breast [38], adrenal, testicular and renal cancers [39]. However, safety data from clinical trials and a recent meta-analysis do not suggest an association between the use of SGLT2i and overall cancer risk [40], although a possible increased risk of bladder cancer has been reported in patients being treated with empagliflozin [40]. On the other hand, tumor cachexia is often associated with glucose intolerance, insulin resistance, and inflammation, which predispose to T2D development [41]. Cancer-related stress (especially due to acute illnesses, recurrent hospitalizations, surgeries, infections, and hemorrhages) can also induce hyperglycemia and worsen inflammation [41]. Finally, diabetes may occur when cancer affects organs involved in glycemic homeostasis, such as the pancreas and liver [4]. In addition to the increased risk of developing cancer, diabetes has been associated with a ∼10% increase in mortality for all cancers (up to 25% for several types of cancer) when compared to the absence of diabetes [42, 43]. As a consequence, while deaths from vascular diseases (which once accounted for more than 50% of the deaths in diabetic patients) have declined, in some countries, cancer has become the leading causes of mortality in people with diabetes [44, 45]. Diabetes may predict a worse prognosis in patients with cancer [42, 46], and more recent findings suggest a key role for poor glycemic control in this scenario [47]. Although only few studies have evaluated the association between glycemic control and survival in patients with both diabetes and cancer [47], several retrospective studies suggest that inadequate glycemic control during cancer follow-up could be associated with poorer tumor response to therapy and survival in patients with diabetes [48, 49]. Unfortunately, in diabetic patients with cancer, oncologists and patients are inclined to prioritize cancer treatment [50, 51] and may accept less stringent glycemic control as a justifiable adverse effect of that treatment [52]. Indeed, cancer treatment is associated with decreased diabetes medication adherence and self-management behaviors such as blood glucose monitoring [53–56]. In addition, several cancer therapies, such as corticosteroids, specific chemotherapies, immune checkpoint inhibitors, and somatostatin analogs, can directly affect glucose homeostasis, thus increasing the risk of hyperglycemia and posing significant difficulties for diabetes management [57, 58]. Despite its importance, the association between glycemic control and cancer outcomes in diabetes patients with cancer remains unsettled and poorly debated. This review article seeks to summarize the available evidence about the possibility that timely treatment of hyperglycemia and improved glycemic control in diabetic patients with cancer can favorably affect cancer outcomes, underlining the importance of careful management of hyperglycemia also in patients with cancer.

## Effects of glycemic control on cancer progression in oncologic patients with diabetes

To date, most studies analyzing the correlation between glycemic control and cancer progression in diabetic patients with cancer have been retrospective in nature and have taken under consideration heterogeneous outcomes to evaluate cancer progression (Table 1). In addition, there is little consistency in how glycemic control is assessed across studies (HbA1c, fasting glucose levels [FG], or random blood glucose [RBG], with different measurement timing and cut-off points) [8]. Above all, it should be mentioned that in patients treated with anti-cancer drugs, HbA1c measurements could be misleading due to interfering non-glycemic factors such as anemia, impaired hematopoiesis, iron, vitamin B12 or folate deficiency, red blood cell transfusion and erythropoiesis-stimulating agent [59]. This makes studies very heterogeneous and difficult to be compared. Moreover, it has been shown that diabetes is associated with more advanced cancer stage and that oncologists might modify anticancer treatments in patients with cancer and diabetes because of increased rates of adverse effects and complications. Despite this, studies analyzing the association between diabetes and mortality from cancer rarely take into account stage at cancer diagnosis or cancer treatments, and this may affect the results [60, 61]. In addition, T2D patients are often obese, implying the need for adequate adjustment of the antineoplastic dosing [62]. Unfortunately, there is a lack of pharmacokinetics data on obese patients for the majority of chemotherapeutic agents, as well as for new cancer targeted therapies and immunotherapy agents [62]. Despite these flaws, most studies suggest that adequate glycemic control may be associated with more favorable neoplastic outcomes, in terms of survival, progression and cancer recurrence. In a recent meta-analysis [63] including twelve studies comprising a total of 9,872 patients with cancer, hyperglycemia was associated with worse overall survival (OS) (Hazard Ratio [HR] 2.05, 95% Confidence Interval [CI] 1.67–2.51) and disease-free survival (DFS) (HR 1.98, 95% CI 1.20–3.27), without any correlation with neoplastic progression-free survival (PFS) (Table 1). The association between hyperglycemia and OS was independent of the method of measuring blood glucose and stage of neoplastic disease. Similarly, a prospective 12-week longitudinal study [64] showed that, in 18 adult patients with T2D and a solid or hematological tumor receiving outpatient intravenous chemotherapy, a good glycemic control (HbA1c < 7.0%) at the onset of cancer therapy may contribute to less adverse events, infections and hospitalizations, and to diminish the number of cases in which a reduction in dosage or an interruption of chemotherapy was necessary (Table 1); however, this study is limited by the low number of enrolled patients. In a ten-year prospective cohort study of 1,298,385 Koreans, a linear trend in cancer-related mortality with increasing FG was observed in patients with a FG > 125 mg/dL compared with those with FG < 90 mg/dL for most cancer sites (in particular, pancreas, liver, and breast in women) [65] (Table 1). However, in this study, glucose testing was done at one time point at baseline, which may not be a reflection of persistent hyperglycemia. Likewise, in an analysis conducted on 820,900 subjects from 97 retrospective studies, it was observed that, as compared with the reference group (FG of 70 to 100 mg/dL), patients with a FG of 126 mg/dL or more exhibit an HR of 1.39 (95% CI, 1.22 to 1.59) for cancer deaths [43]. A HR for cancer death of 1.05 (95% CI 1.03–1.06) for every 1 mmol/l increase in glucose levels above 100 mg/dL was also reported [43] (Table 1). However, in this analysis, the glucose measurements were not conducted in the proximity of cancer diagnosis and thus do not reflect the levels of glycemic control at that time (it is possible that those measurements may be more relevant for assessing cancer risk rather than outcomes). In addition, this study looked at all cancer related deaths instead of death from specific types of cancer [66]. Several observational studies also suggest that inadequate glycemic control, in the pre- or postoperative of a surgical cancer treatment, significantly worsens clinical outcomes of cancer and increases the risk of cancer recurrence [50]. Furthermore, in diabetic patients with cancer, poor glycemic control could exacerbate the risk of postoperative or post-chemotherapy infections and increase the perception of pain and fatigue often experienced by oncologic patients [50]. On the contrary, in a retrospective study conducted on 7916 individuals with incident cancers and concurrent diabetes [66], higher glucose and HbA1c levels within 6 months prior to cancer diagnosis was not associated with worse OS following cancer diagnosis. Interestingly, among diabetic patients treated with insulin, increased survival with increasing serum glucose was observed, most prominent for bladder cancer (HR 0.91, 95% CI 0.84–0.99, per 1 mmol/l increase) [66] (Table 1). Similarly, in a cross-sectional study conducted on 244 diabetic or prediabetic patients with breast, gastrointestinal, gynecological or lung cancer, adequate glycemic control was not associated with the severity of tumor-related symptoms or with the patient’s quality of life [67] (Table 1). Very few studies have analyzed the importance of glycemic control in diabetic patients with terminal cancers. However, all such studies agree that, at this stage of the oncologic disease, glycemic control may play a role in symptom management and prolonging survival [8, 68]. Hypoglycemia has also been associated with a poorer prognosis in patients with diabetes and cancer [69]. In a prospective cohort analysis of 1209 participants with diagnosed diabetes from the Atherosclerosis Risk in Communities study, severe hypoglycemia was significantly associated with cancer mortality (HR 2.49, 95% CI 1.46–4.24) [70] (Table 1). Severe hypoglycemia is very likely an indicator of frailty, which is causally linked to poor cancer survival [69]. In the following sections, we will summarize the available evidence about the correlation between glycemic control and cancer outcomes in diabetic patients with cancers most linked to diabetes (bladder, breast, colon/rectum, endometrium, liver, pancreas, and prostate). To the best of our knowledge, no studies have explored such a correlation in diabetic patients with gallbladder or biliary tract cancer.

**Table 1:** Evidence on the association between glycemic control and cancer outcomes in oncologic patients with diabetes

Cancer	Type of study	Glycemic control (evaluation method)	Cancer outcome	References
Various cancers	Review and meta-analysis	Various methods	↑ Survival and DFS, = PFS	[63]
Various cancers	Prospective	HbA1c	↓ Adverse events, reduction or interruption of chemotherapy	[64]
Various cancers	Prospective	FG	↓ Mortality	[65]
Various cancers	Analysis of 97 retrospective studies	FG	↓ Cancer death	[43]
Various cancers	Retrospective	HbA1c 6 months before cancer diagnosis	= OS; ↓ survival in patients with bladder cancer and treated with insulin	[66]
Various cancers	Cross-sectional	HbA1c during chemotherapy	= Severity of symptoms	[67]
Various cancers	Prospective	HbA1c	↓ Mortality	[70]

↑ adequate glycemic control increases the probability of the indicated outcome, ↓ adequate glycemic control reduces the probability of the indicated outcome, = adequate glycemic control has no effect on the indicated outcome, *DFS* disease-free survival, *FG* fasting glucose levels, *HbA1c* glycated hemoglobin, *OS* overall survival, *PFS* progression-free survival

### Bladder

Diabetes has been associated with higher incidence and poor prognosis of bladder cancer [71]. Furthermore, poor glycemic control results in increased oxidative stress and inflammation, which are thought to play a negative effect on bladder cancer prognosis [71]. A retrospective study conducted on 287 patients with non-muscle invasive bladder cancer (61 with DM and 266 without DM) revealed higher recurrence rate and worse recurrence-free survival (RFS) in patients with HbA1c ≥ 7% [71] (Table 2). Of note, the use of metformin or thiazolidinediones, which may influence bladder cancer outcomes, was not associated with RFS [71]. Similarly, a retrospective study on 538 patients with upper urinary tract urothelial carcinoma, has demonstrated that poor glycemic control (HbA1c ≥ 7%) is associated with increased risk of subsequent bladder cancer recurrence (HR 2.10, 95% CI 1.14–3.88) [72] (Table 2). Likewise, in a cohort of 645 patients with non-muscle invasive bladder cancer analyzed retrospectively, diabetic patients with a HbA1c ≥ 7% demonstrated a higher rate of cancer progression [73]. Kaplan–Meier analysis showed that poor baseline glycemic control and post-operative glycemic control were associated with lower PFS rate [73] (Table 2). Of note, use of metformin had no impact on the recurrence and progression of cancer [73]. Finally, in a cohort of 251 patients who underwent transurethral resection for non-muscle invasive bladder cancer analyzed retrospectively, it was observed that patients with HbA1c ≥ 7% exhibited a significantly higher rate of multiplicity and tumor grade [74] (Table 2). These results underscore the need for intensive glycemic control and close follow-up for diabetic patients with bladder cancer.

**Table 2:** Evidence on the association between glycemic control and bladder cancer outcomes in oncologic patients with diabetes

Cancer	Type of study	Glycemic control (evaluation method)	Cancer outcome	References
Non-muscle invasive bladder cancer	Retrospective	HbA1c	↓ Recurrence and ↓ RFS	[71]
Bladder cancer after upper urinary tract urothelial carcinoma	Retrospective	HbA1c	↓ Recurrence	[72]
Non-muscle invasive bladder cancer	Retrospective	Pre- and post-surgical HbA1c	↓ Progression and ↑ PFS	[73]
Non-muscle invasive bladder cancer	Retrospective	HbA1c	↓ Cancer multiplicity and grade	[74]

↑ adequate glycemic control increases the probability of the indicated outcome, ↓ adequate glycemic control reduces the probability of the indicated outcome, = adequate glycemic control has no effect on the indicated outcome, *HbA1c* glycated hemoglobin, *PFS* progression-free survival, *RFS* recurrence-free survival

### Breast

Diabetes is a known risk factor for the development of breast cancer. Approximately 10 to 20% of all postmenopausal women with breast cancer of any stage or receptor subtype have coexisting T2D [75]. Studies investigating the effects of glycemic control on breast cancer outcomes have yielded mixed results. In a recent prospective study conducted on 620 patients with breast cancer with a follow-up of approximatively 6 years, the HRs and 95% CI for mortality rate was higher in patients with inadequate glycemic control prior to cancer diagnosis compared with patients with glycemic control at target (HR 1.40, 95% CI 1.00–1.96) [47] (Table 3). In a retrospective study conducted on 243 patients with non-metastatic breast cancer with or without diabetes receiving neoadjuvant or adjuvant cytotoxic chemotherapy, higher utilization of emergency departments and higher frequency of unplanned inpatient admissions were detected in patients with HbA1c > 7% compared to those with HbA1c ≤ 7% [76]. In addition, patients with HbA1c > 7% showed a shorter time until the first emergency department visit and experienced more adverse events compared to those with HbA1c ≤ 7% [76]. Moreover, the percentage of documented infections was higher among oncologic patients with HbA1c > 7% compared to those without diabetes [76] (Table 3). Chang YL et al. have retrospectively analyzed 2812 women with early breast cancer (145 with and 2667 without diabetes), demonstrating the existence of a relationship between glycemic control and breast cancer prognosis in women with diabetes: specifically, a mean HbA1c > 9% in breast cancer women was associated with a 3.65-fold (95% CI 1.13–11.82) higher risk of all-cause mortality, including cancer-specific mortality, while patients with well-controlled diabetes (HbA1c < 7%) had comparable survival to individuals without diabetes [77]. In addition, lower HbA1c (< 7%) may be associated with more favorable breast cancer progression outcomes [77] (Table 3). Similarly, a substudy of the Women’s Healthy Eating and Living (WHEL) study found that hyperglycemia (HbA1c ≥ 7%) was statistically significantly associated with reduced OS but not with DFS (HR 1.26, 95% CI 0.78–2.02) in 3,003 individuals with early breast cancer [78]. In addition, the risk of all-cause mortality was twice as high in individuals with a HbA1c ≥ 7%, suggesting that good glycemic control may be associated with better breast cancer prognosis [78] (Table 3). In contrast, Cheung YMMM et al. retrospectively compared 244 patients with diagnosis of metastatic breast cancer with diabetes to 244 patients with diagnosis of metastatic breast cancer without diabetes [79]. OS was found not to differ among patients with good glycemic control (RBG ≤ 180 mg/dL or HbA1c ≤ 7%) compared to those with poor control [79]. However, poor glycemic control was associated with greater mortality in longer-term cancer survivors [79] (Table 3). Interestingly, at 5 years, there was a trend toward a better OS among patients who received metformin monotherapy compared to those who received metformin in addition to other glucose-lowering agents, as well as those who did not received metformin [79]. Moreover, a retrospective cohort study including 82 patients with breast cancer found that OS was not statistically different among participants with HbA1c < 6.5% and ≥ 6.5% [80] (Table 3). It should be noted that several of these studies did not adjust for confounders such as receptor subtype, cancer stage, or medication regimen and usually relied on a single HbA1c measurement to define glycemic control [79].

**Table 3:** Evidence on the association between glycemic control and breast cancer outcomes in oncologic patients with diabetes

Cancer	Type of study	Glycemic control (evaluation method)	Cancer outcome	References
Breast cancer	Prospective	HbA1c before cancer diagnosis	↓ Mortality	[47]
Non-metastatic breast cancer	Retrospective	HbA1c	↓ ED visits, unplanned inpatients admission, time until the first ED visit, infections	[76]
Early breast cancer	Retrospective	HbA1c	↓ Progression and mortality	[77]
Early breast cancer	Retrospective	HbA1c	↑ OS, ↓ mortality, = DFS	[78]
Metastatic breast cancer	Retrospective	HbA1c and RBG	= OS at five years, ↑ OS in long-term survivors	[79]
Breast cancer	Retrospective	HbA1c	= OS	[80]

↑ adequate glycemic control increases the probability of indicated outcome, ↓ adequate glycemic control reduces the probability of the indicated outcome, = adequate glycemic control has no effect on the indicated outcome, *DFS* disease-free survival, *ED* emergency department, *HbA1c* glycated hemoglobin, *OS* overall survival, *RBG* random blood glucose

### Colon-rectum

Diabetes is also a known risk factor for the development of colorectal cancer. Several studies have investigated the effects of glycemic control on colorectal cancer outcomes. In a recent prospective study conducted on 774 patients with colorectal cancer with a follow-up of approximatively 6 years, the HRs and 95% CI for mortality was higher in patients with glycemic control not at target prior to cancer diagnosis compared with patients at target (HR 1.45, 95% CI 1.12–1.88) [47] (Table 4). Similarly, in 741 patients with colon cancer analyzed retrospectively, the concomitant presence of uncontrolled diabetes (HbA1c ≥ 8%) resulted in significantly shorter OS and higher mortality compared to well-controlled diabetic patients [81] (Table 4). Similar results have been obtained in a case–control study involving 224 patients with colorectal cancer and 112 controls [82]. Elevated HbA1c levels showed a negative prognostic value both in terms of PFS (HR = 1.24) and OS (HR = 1.36) after adjustment for major confounders [82] (Table 4). Likewise, Siddiqui AA et al. have shown that, in 155 patients with T2D and colorectal cancer compared to 114 control patients who had colorectal cancer without T2D, poor glycemic control (HbA1c ≥ 7.5%) was associated with a more clinically aggressive cancer course (advanced cancer stage, younger age of cancer presentation, and poorer 5-year survival) [83] (Table 4). To the best of our knowledge, only one study has shown conflicting data [84]. It is a retrospective cohort study conducted on 210 patients with advanced colorectal cancer and concomitant T2D, which demonstrated that the OS of patients with a baseline FG ≤ 126 mg/dL was not significantly prolonged compared to patients with a baseline FG > 126 mg/dL [84] (Table 4). These discordant results could be attributed the fact that in this study, unlike the others, patients at an advanced stage of colorectal cancer were enrolled, in whom the OS may have been already compromised.

**Table 4:** Evidence on the association between glycemic control and colorectal cancer outcomes in oncologic patients with diabetes

Cancer	Type of study	Glycemic control (evaluation method)	Cancer outcome	References
Colorectal cancer	Prospective	HbA1c before cancer diagnosis	↓ Mortality	[47]
Colon	Retrospective	HbA1c	↑ OS and ↓ mortality	[81]
Colorectal cancer	Case–control	HbA1c	↑ Survival and PFS	[82]
Colorectal cancer	Retrospective	HbA1c	↓ Aggressiveness, ↑ 5-year survival and ↑ age at onset	[83]
Advanced colorectal cancer	Retrospective	FG	= OS	[84]

↑ adequate glycemic control increases the probability of the indicated outcome, ↓ adequate glycemic control reduces the probability of the indicated outcome, = adequate glycemic control has no effect on the indicated outcome, FG fasting glucose levels, HbA1c glycated hemoglobin, OS overall survival, PFS progression-free survival

### Endometrium

Several studies have demonstrated that patients with diabetes have an increased risk of endometrial cancer, and retrospective studies have shown that patients with endometrial cancer and coexisting diabetes have worse survival than those without [85]. In a recent retrospective study conducted on 96 women with endometrial cancer (48 with, 48 without diabetes), no statistical difference in OS was found for patients with diabetes who achieved glycemic control (mean FG value < 126 mg/dL during the year after cancer diagnosis) versus those who did not [85] (Table 5). Interestingly, Raffone A et al. [86] have reviewed and meta-analyzed the role of glycemic control in the progression of endometrial hyperplasia to endometrial cancer, demonstrating that adequate glycemic control may be required in women with endometrial hyperplasia in order to reduce the risk of imminent progression in endometrial cancer (Table 5). Finally, Stevensen EE et al. [87], analyzing 82 patients with endometrial cancer who underwent surgical staging and had HbA1c drawn within 3 months before surgery, have demonstrated that high preoperative HbA1c had a trend toward a higher stage of endometrial cancer at the time of diagnosis (Table 5).

**Table 5:** Evidence on the association between glycemic control and endometrial cancer outcomes in oncologic patients with diabetes

Cancer	Type of study	Glycemic control (evaluation method)	Cancer outcome	References
Endometrial cancer	Retrospective	FG	= OS	[85]
Occult endometrial cancer in endometrial hyperplasia	Review and meta-analysis	Various methods	↓ Progression	[86]
Endometrial cancer	Retrospective	HbA1c and FG within 3 months before surgery	↓ Cancer stage at diagnosis	[87]

↑ adequate glycemic control increases the probability of the indicated outcome, ↓ adequate glycemic control reduces the probability of the indicated outcome, = adequate glycemic control has no effect on the indicated outcome, FG fasting glucose levels, HbA1c glycated hemoglobin, OS overall survival

### Liver

Although there is ample evidence that diabetes is associated with increased risk of liver cancer [6, 7, 10, 88–90], to the best of our knowledge only one study has analyzed the role of glycemic control on liver cancer outcomes. In this study, 100 patients who underwent curative resection for solitary hepatitis C virus-related hepatocellular carcinoma (26 with diabetes and 74 without) were analyzed [91]. DFS rate was 66 and 27% at 3 years in patients with normal postoperative HbA1c level (< 6.5%) and elevated postoperative HbA1c level (≥ 6.5%), respectively [91]. In addition, multivariate analysis showed that poor glycemic control (HbA1c ≥ 6.5%) was associated with postoperative tumor recurrence in patients with diabetes [91].

### Pancreas

As for liver cancer, evidence suggests that diabetes is associated with increased risk of pancreatic cancer [6, 7, 10, 92, 93]. However, in the case of pancreatic cancer it is difficult to distinguish whether it is the glycemic control that influences the cancer outcomes or vice versa, as pancreatic cancer and its treatment (pharmacological or surgical) may induce hyperglycemia [94]. Several studies have analyzed the association between glycemic control and pancreatic cancer outcomes (Table 6). Alpertunga I et al. [95] have studied 73 patients with advanced pancreatic ductal adenocarcinoma receiving chemotherapy. They found that a 3-month average RBG ≤ 120 mg/dL predicted for improved OS compared to RBG > 120 mg/dL (19 vs. 9 months; HR = 0.37) in both patients with and without diabetes [95] (Table 6). There were no differences in OS between metformin or insulin users and non-users [95]. In another retrospective study conducted on 417 patients (88 with diabetes) with pancreatic neuroendocrine neoplasms undergoing surgical resection, patients with dysglycemia (FG ≥ 140 mg/dL or HbA1c ≥ 6.5%) had greater rates of metastasis [96]. In addition, preoperative dysglycemia was associated with impaired OS (HS 1.57, 95% CI 1.01–2.46) and RFS (HR 1.78, 95% CI 1.01–3.12], regardless of the presence of diabetes [96] (Table 6). Similarly, elevated preoperative HbA1c has been associated with failure to complete anti-cancer therapy or surgery and a trend for increased risk of metastatic progression in 123 patients with localized pancreatic cancer [97] (Table 6). Finally, in a retrospective study of 52 patients with pancreatic tumors who underwent total pancreatectomy, elevated postoperative FG levels were significantly associated with complications after surgery [98]. In addition, postoperative HbA1c levels over 7% were identified as one of the independent risk factors for tumor recurrence (HR 2.655, 95% CI 1.299–5.425). Patients with postoperative HbA1c levels over 7% had poorer OS than those with HbA1c levels less than 7% (HR 3.212, 95% CI 1.147–8.999) [98] (Table 6).

**Table 6:** Evidence on the association between glycemic control and pancreatic cancer outcomes in oncologic patients with diabetes

Cancer	Type of study	Glycemic control (evaluation method)	Cancer outcome	References
Advanced pancreatic ductal adenocarcinoma	Retrospective	RBG	↑ OS	[95]
Pancreatic neuroendocrine neoplasms	Retrospective	HbA1c or FG	↑ OS, ↓ RFS and metastasis	[96]
Localized pancreatic cancer	Retrospective	HbA1c before any therapy and after neoadjuvant therapy, before surgery	↑ Completion of therapies or surgery, ↓ metastasis	[97]
Pancreatic cancer	Retrospective	FG/HbA1c after total pancreatectomy	↓ Surgical complications, recurrence, ↑ OS	[98]

↑ adequate glycemic control increases the probability of the indicated outcome, ↓ adequate glycemic control reduces the probability of the indicated outcome, = adequate glycemic control has no effect on the indicated outcome, *FG* fasting glucose levels, *HbA1c* glycated hemoglobin, *OS* overall survival, *RBG* random blood glucose, *RFS* recurrence-free survival

### Prostate

As stated above, the relationship between prostate cancer and diabetes is unique, since it is the only cancer where diabetes appears to be protective [13]. The underlying cause of this protective role is not fully understood; however, some mechanisms have been proposed [99]. Specifically, elevated circulating levels of androgen have been suggested as risk factor for prostate cancer and could work as tumor growth factors. As a consequence, the reduced levels of androgen that occur in diabetes may represent a protective factor against prostate cancer [99]. Similarly, type 1 diabetes and long-lasting type 2 diabetes with prevalent secretory dysfunction are associated with insulin depletion and decreased IGF-1 signaling which could further explain the protective role of diabetes on prostate cancer [99]. Despite the protective role if diabetes on the risk of prostate cancer, several studies suggest that prostate cancer patients with diabetes and poor glycemic control may have increased risk of biologically aggressive cancer (Table 7). In a recent prospective study conducted on 438 patients with prostate cancer with a follow-up of approximatively 6 years, the HRs and 95% CI for mortality rate was higher in patients with inadequate glycemic control prior to cancer diagnosis compared with patients with adequate glycemic control (HR 1.39, 95% CI 0.98–1.98) [47] (Table 7). A retrospective study conducted on 831 patients with prostate cancer with or without preexisting diabetes showed that mean HbA1c levels ≥ 9% had significantly increased risk for all-cause and non-prostate cancer mortality (HR 3.09, 95% CIs 1.15–8.32 and HR 5.49, 95% CIs 1.66–18.16, respectively), but not for prostate cancer-specific mortality (HR 1.03, 95% CIs 0.13–8.44) compared with the non-diabetes group [100] (Table 7). These results were confirmed also after adjusting for metformin use [100]. Nik-Ahd F et al. [49] have retrospectively reviewed data regarding 1,409 men with prostate cancer undergoing radical prostatectomy (710 with diabetes) with a median follow-up of 6.8 years. They found that a higher HbA1c value was associated with metastasis (HR 1.21, 95% CI 1.02–1.44) and castration-resistant prostate cancer (HR 1.27, 95% CI 1.03–1.56) [49]. Although not statistically significant, there were trends between higher HbA1c and risk of prostate cancer-specific mortality and all-cause mortality [49] (Table 7). In addition, Lee H et al. [101] demonstrated that poorer glycemic control (HbA1c levels ≥ 6.5% within the 6 months preceding radical prostatectomy) was significantly related with high cancer aggressiveness and biochemical recurrence-free survival in 746 prostate cancer patients with (n = 209) or without (n = 537) diabetes (Table 7). Meanwhile, metformin use was not associated with biochemical recurrence-free survival [101]. Likewise, in a retrospective study conducted on 731 men with prostate cancer (338 with a history of diabetes) poor glycemic control was associated with a higher risk of high-grade prostate cancer detection [102] (Table 7). Similar results have been demonstrated by Kim HS et al. [103], showing that men with higher HbA1c levels presented with more biologically aggressive prostate cancer at radical prostatectomy, although HbA1c levels were not significantly related to risk of biochemical recurrence [103] (Table 7). In addition, in patients with prostate cancer, average glycemia during chemotherapy was significantly associated with overall severe toxicity [104] (Table 7). Finally, Hong SK et al. [105] have demonstrated that higher HbA1c levels (≥ 6.5%) were associated with a significantly higher rate of extraprostatic extension of tumor and higher cancer aggressiveness comparted to HbA1c levels < 6.5% (Table 7). In contrast with these studies, Joentausta RM et al. [106] found that glycemic control after radical prostatectomy was not associated with cancer recurrence and short-term mortality in 1,314 men who underwent radical prostatectomy (Table 7). Importantly, duration, and dose of anti-diabetes medication use had no effect on cancer survival [106].

**Table 7:** Evidence on the association between glycemic control and prostate cancer outcomes in oncologic patients with diabetes

Cancer	Type of study	Glycemic control (evaluation method)	Cancer outcome	References
Prostate cancer	Prospective	HbA1c before cancer diagnosis	↓ Mortality	[47]
Prostate cancer	Retrospective	HbA1c	↓ Mortality, = cancer-related mortality	[100]
Prostate cancer	Retrospective	HbA1c before radical prostatectomy	↓ Metastasis and resistance to castration	[49]
Prostate cancer	Retrospective	HbA1c within 6 months before radical prostatectomy	↓ Recurrence, ↓ BCR-free survival	[101]
Prostate cancer	Retrospective	HbA1c	↓ High-grade cancer	[102]
Prostate cancer	Retrospective	HbA1c before radical prostatectomy	↓ Aggressiveness, = BCR	[103]
Prostate cancer	Retrospective	FG during chemotherapy	↓ Chemotherapy toxicity	[104]
Prostate cancer	Retrospective	HbA1c	↓ Aggressiveness and extraprostatic extension	[105]
Prostate cancer	Retrospective	FG/HbA1c after radical prostatectomy	= Recurrence and short-term mortality	[106]

↑ adequate glycemic control increases the probability of indicated outcome, ↓ adequate glycemic control reduces the probability of the indicated outcome, = adequate glycemic control has no effect on the indicated outcome, *BCR* biochemical recurrence, *FG* fasting glucose levels, *HbA1c* glycated hemoglobin

## Conclusions

Growing evidence suggests that patients with diabetes are characterized by an increased risk of developing different types of cancer and reduced survival after cancer diagnosis [6, 7]. In particular, diabetes increases the risk of developing bladder, breast, colorectal, endometrial, gallbladder, liver, and pancreatic cancers [6, 7], while reducing the risk of developing prostate cancer [13]. While diabetes and cancer share several common risk factors, and therefore the probability of their occurrence in the same patient is high [12, 14], growing evidence suggests that diabetes and cancer could cause each other with distinct mechanisms. Indeed, hyperinsulinemia, hyperglycemia, and chronic low-grade inflammation may represent the main pathophysiological factors underlying this correlation. Research has also shown that diabetes may predict a worse prognosis in patients with cancer [42, 46], with more recent findings suggesting an important role for poor glycemic control [47]. Nevertheless, only few studies have evaluated the association between glycemic control and survival in patients with both cancer and diabetes, yielding mixed results [47]. Most, but not all, studies analyzed in this review suggest that a good glycemic control may favorably influence cancer outcomes (in terms of survival, progression, recurrence, aggressiveness, and response to therapy). However, few other studies show no effect of glycemic control on cancer outcomes, while no studies suggest that a good glycemic control could have negative effects (except, perhaps, in terms of quality of life). Altogether, these results endorse the importance of multidisciplinary diabetes management in oncologic patients. Indeed, there is a growing need of interdisciplinary competence and coordination between diabetologists and oncologists to better manage patients with both diabetes and cancer, since the coexistence of the two diseases poses significant challenges for patients and health care providers [107]. It should be highlighted that most of the studies analyzing the correlation between glycemic control and cancer progression face several methodological concerns. Most of them are retrospective in nature, while prospective studies could provide better-quality evidence and the possibility of adjusting the results for more confounding factors. In addition, these studies take under consideration heterogeneous outcomes to evaluate cancer progression (OS, PFS, RFS, and others), and there is little consistency in how glycemic control is measured across studies (HbA1c, FG, or RBG, with different measurement timing and cut-off points) [8]. Moreover, most of these studies do not take into account several important factors that may affect cancer outcomes, such as population ethnicity, age and stage at cancer diagnosis, nutritional status, or cancer treatments [60, 61, 94]. Studying the association between diabetes, glycemic control, cancer risk, and cancer outcomes is further complicated by evidence that anti-diabetes drugs themselves may influence the risk of cancer development and progression (reviewed in [10]). In particular metformin, for its preventive effect on a fair number of cancers [108], and pioglitazone or empagliflozin, for their possible association with a higher risk of bladder cancer [35, 36, 40, 109, 110]. Nevertheless, in only a few studies among those analyzed in this review, data are adjusted for anti-diabetes therapy or the use of metformin. Information on anti-diabetes therapy is often lacking. Ultimately, despite its importance, the association between glycemic control and cancer outcomes in diabetic patients with cancer remains unsettled and poorly debated. Although there are good reasons to believe that a good glycemic control may favorably influence cancer outcomes, further prospective studies, including larger patients’ cohorts and addressing all relevant methodological issues, are needed.

## Data Availability

Not applicable.
